# Birt-Hogg-Dube syndrome prospectively detected by review of chest computed tomography scans

**DOI:** 10.1371/journal.pone.0170713

**Published:** 2017-02-02

**Authors:** Hye Jung Park, Chul Hwan Park, Sang Eun Lee, Geun Dong Lee, Min Kwang Byun, Sungsoo Lee, Kyung-A Lee, Tae Hoon Kim, Seong Han Kim, Seo Yeon Yang, Hyung Jung Kim, Chul Min Ahn

**Affiliations:** 1 Pulmonary Division, Department of Internal Medicine, Gangnam Severance Hospital, Yonsei University College of Medicine, Seoul, Korea; 2 Department of Radiology, Gangnam Severance Hospital, Yonsei University College of Medicine, Seoul, Korea; 3 Department of Dermatology, Gangnam Severance Hospital, Yonsei University College of Medicine, Seoul, Korea; 4 Department of Thoracic and Cardiovascular Surgery, Gangnam Severance Hospital, Yonsei University College of Medicine, Seoul, Korea; 5 Department of Laboratory Medicine, Gangnam Severance Hospital, Yonsei University College of Medicine, Seoul, Korea; Universita degli Studi di Napoli Federico II, ITALY

## Abstract

**Purpose:**

Birt-Hogg-Dube syndrome (BHD) is a rare disorder caused by mutations in the gene that encodes folliculin (*FLCN*) and is inherited in an autosomal dominant manner. BHD is commonly accompanied by fibrofolliculomas, renal tumors, multiple pulmonary cysts, and spontaneous pneumothorax. The aim of this study was to detect BHD prospectively in patients undergoing chest computed tomography (CT) scans and to evaluate further the characteristics of BHD in Korea.

**Methods:**

We prospectively checked and reviewed the chest CT scans obtained for 10,883 patients at Gangnam Severance Hospital, Seoul, Korea, from June 1, 2015 to May 31, 2016. Seventeen patients met the study inclusion criteria and underwent screening for *FLCN* mutation to confirm BHD. We analyzed the characteristics of the patients confirmed to have BHD and those for a further 6 patients who had previously been described in Korea.

**Results:**

Six (0.06%) of the 10,883 patients reviewed were diagnosed with BHD. There was no difference in demographic or clinical features between the patients with BHD (n = 6) and those without BHD (n = 11). Pneumothorax was present in 50% of the patients with BHD but typical skin and renal lesions were absent. The maximum size of the cysts in the BHD group (median 39.4 mm; interquartile range [IQR] 11.4 mm) was significantly larger than that in the non-BHD group (median 15.8 mm; IQR 7.8 mm; *P* = 0.001). Variable morphology was seen in 100.0% of the cysts in the BHD group but in only 18.2% of the cysts in the non-BHD group (*P* = 0.002). Nine (95%) of the total of 12 Korean patients with BHD had experienced pneumothorax. Typical skin and renal lesions were present in 20.0% of patients with BHD.

**Conclusions:**

Our findings suggest that BHD can be detected if chest CT scans are read in detail.

## Background

Birt–Hogg–Dube syndrome (BHD) is a rare inherited autosomal dominant disease characterized by multiple pulmonary cysts and by skin and renal lesions. BHD is caused by loss-of-function mutations in germline folliculin, encoded by *FLCN*, which has been mapped to chromosome 17p11.2 [1]. Folliculin is thought to function as a tumor suppressor that is associated with the mammalian target of rapamycin (mTOR) pathway [2]. Defects in folliculin affect the cytoskeleton, disrupt the extracellular matrix, and suppress cellular proliferation [3]. *FLCN* is expressed in the skin, distal nephron of the kidney, and the pneumocytes of the lung [4]. Therefore, patients with BHD and *FLCN* mutations present with multiple lung cysts, cutaneous manifestations including fibrofolliculoma, trichodiscoma, acrochordon, and perifollicular fibroma, and various renal tumors.

BHD was first described in the 1970s [2]. In the USA and Europe, typical skin lesions are found in more than 80% of patients with BHD. Typical renal lesions are noted in about 15–35% of patients [5,6]. However, recent studies have shown that these typical skin and renal lesions are less frequently present in Asian patients, including those from China, Japan, and Taiwan [7–9]. For example, in China, Ding et al. have reported that typical skin and renal lesions were present in 20% and 0%, respectively, of affected members in 8 families representing 40 individuals) [7].

Until now, only 430 families with BHD have been reported worldwide, with the largest containing 36 affected members [10,11]. Only 6 cases of BHD have been reported in Korea [12–15]. Given the rarity of this syndrome, only case reports and case reviews of BHD have been reported to date. To our knowledge, no prospective study has attempted to diagnose BHD syndrome. Further, there are no literature reviews on BHD in Korea.

The purpose of the present study was to detect BHD prospectively in patients undergoing chest computed tomography (CT) and to analyze the characteristics of BHD in Korea.

## Methods

### Ethics approval and consent to participate

This study was approved by the institutional review board of the Gangnam Severance Hospital, Yonsei University Health System (approval number: 3-2015-0122).

### Diagnostic criteria

BHD was diagnosed according to the previous literature [1]. Patients were required to fulfill one major or 2 minor criteria for the diagnosis of BHD. The major inclusion criteria were as follows: 1) at least 5 fibrofolliculomas or trichodiscomas, of which at least one should be histologically confirmed, with adult onset; and 2) evidence of pathogenic *FLCN* germline mutation. The minor inclusion criteria were as follows: 1) bilaterally located multiple basal lung cysts, with no other apparent cause, with or without spontaneous primary pneumothorax; 2) renal cancer with early onset (< 50 years), or with multifocal or bilateral location, or with mixed chromophobe and oncocytic histology; and 3) a first-degree relative with BHD. In the present study, typical skin and renal lesions were defined as described above.

### Inclusion and exclusion criteria for study enrolment

Inclusion criteria comprised the following: 1) age older than 19 years and 2) chest CT scan results consistent with BHD [1,16]. We defined pulmonary cysts as air-filled spaces with a sharply demarcated thin wall less than 3 mm in thickness [17]. The upper and lower zones were divided at the level of the tracheal carina along the axial direction with a craniocaudal distribution. Multiple cysts located predominantly in the lower zones were considered to be consistent with BHD. Exclusion criteria were as follows: 1) cysts located predominantly in the upper zones; 2) cysts located in one lung only; and 3) cysts with characteristics that were more consistent with other cystic lung diseases, such as lymphangioleiomyomatosis.

### Study group

**Fig 1:**
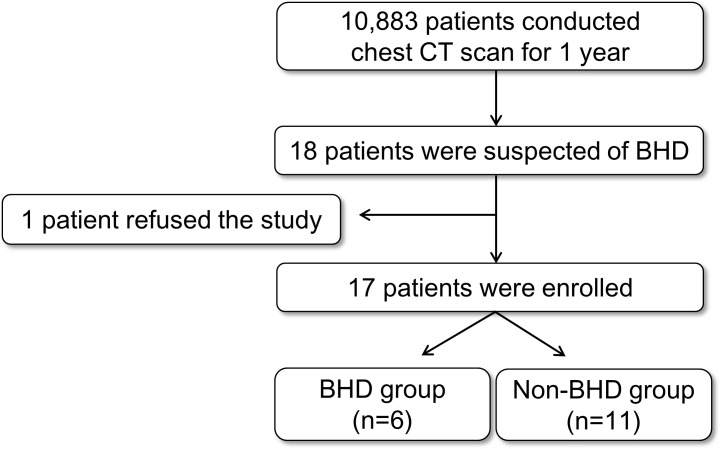
Study flow chart. ***CT***, computed tomography; ***BHD***, Birt–Hogg–Dube syndrome.

A total of 10,883 patients were identified as having undergone chest CT scans at Gangnam Severance Hospital, Seoul, Korea, between June 1, 2015 and May 31, 2016. On the basis of a careful review of the chest CT scans, 18 patients were suspected to have BHD. One patient refused to participate in the study, leaving 17 patients who were able to be enrolled in this cohort study. After an *FLCN* gene study to confirm BHD, we classified these patients into a BHD group (positive for *FLCN* mutation) or a non-BHD group (negative for *FLCN* mutation) as shown in Fig 1.

### Chest CT scan protocol

CT scans were performed using one of three scanners: a 16-slice multidetector CT (MDCT) scanner (Somatom Sensation 16; Siemens Medical Solutions, Erlangen, Germany), a 64-slice MDCT scanner (Somatom Sensation 64; Siemens Medical Solutions), or a 128-slice MDCT scanner (Somatom Definition AS+; Siemens Medical Solutions). Scanning was performed in the supine position from the lung apices to the level of the adrenal glands during inspiration. After acquiring the scout image to determine the field of view, conventional CT scanning was performed without contrast enhancement using a helical technique, with a 3 mm or 5 mm reconstruction interval in the mediastinal window setting. The exposure parameters for the CT scans were as follows: 80–100 kVp, 50–130 mA, slice thickness, 1 mm or 5 mm, and a reconstruction increment of 3 mm or 5-mm. Image reconstruction for conventional CT scans was performed on the scanner’s workstation. All CT images were retrieved on an image archiving and communication system (Centricity 2.0; GE Medical Systems, Mt Prospect, IL, USA) and then analyzed using the mediastinal window settings (level, 50 HU; width, 400 HU).

### Characteristics of chest CT

Two radiologists (T.H.K and C.H.P, with over 20 and 9 years’ experience in chest radiology interpretation, respectively) assessed the CT images. The medial and lateral zones divided the lung field into an inner and outer half; axial distribution (as central, peripheral, or evenly distributed). Involvement of the costophrenic angle (CPA) was assessed with regard to the relationship with the visceral pleura (including the interlobular fissure) and were classified into two groups based on whether the cyst bordered the visceral pleura. A multiseptated cyst was defined as at least one further cyst with multiseptation. Variable morphology was defined as being present when patients had all of the following types of cysts: “round-shaped” (a smooth-surfaced and symmetric cyst with a diameter ratio of long to short axes ≤ 1.1); “oval-shaped” (a smooth-surfaced and symmetric cyst with a diameter ratio of long to short axes > 1.1); and “irregular-shaped” (a cyst with a shape other than round or oval) [18].

### FLCN mutation study

We confirmed the presence of BHD based on the results of *FLCN* mutation screening as described elsewhere [12, 15]. Genomic DNA was extracted using an Easy-DNA™ Kit (Invitrogen, Carlsbad, CA, USA) from whole blood samples collected in EDTA-containing tubes. The concentration and quality of genomic DNA were evaluated by Nanodrop (ND-1000, Thermo Scientific, Wilmington, DE, USA). Primers designed to amplify *FLCN* exons 4–14 and their flanking introns were used to perform PCR amplification. The polymerase chain reaction (PCR) products were then purified using a QIA-quick Gel Extraction Kit (Qiagen, Dusseldorf, Germany) and directly cycle-sequenced using the relevant PCR primers and a Big Dye Terminator Cycle Sequencing Ready Reaction Kit (Applied Biosystems, Foster City, CA, USA). These sequences were then compared with the reference sequence using Sequencher software (Gene Codes, Ann Arbor, MI, USA). Pathogenic variants were detected by Sanger sequencing followed by multiple ligation probe amplification to confirm that there were no large deletions in *FLCN* in subjects who were not detected to have pathogenic variants.

### Other tests

Abdominal sonography or abdominal CT scans were performed to identify renal lesions. Pulmonary function tests, including forced volume capacity (FVC), forced expiratory volume in 1 second (FEV_1_), and FEV_1_/FVC. were performed to determine whether pulmonary function was impaired using commercially available equipment (MS-IOS; Masterlab-IOS, Jaeger, Wurzburg, Germany). All subjects attended a consultation with a dermatologist (S.E.L) who carefully performed a full body skin examination to check for skin manifestations. Any lesion suspected of being associated with BHD was biopsied for pathological analysis. Routine urine analysis was performed.

### Analysis of Korean BHD patients

To analyze the characteristics of BHD in Korea, we combined the findings for the 6 newly diagnosed patients in the present study with those for the 6 Korean patients with BHD previously described in the literature [12–15].

### Statistical analysis

The continuous patient variables (Table 1) were compared according to BHD status using the *t*-test because they were normally distributed throughout the patient cohort, as defined by the Kolmogorov-Smirnov and Shapiro-Wilk tests. Among the CT characteristics (Table 2), the minimum and maximum size of cysts were not normally distributed as defined by the same tests so were assessed using the Mann-Whitney *U* test. Fisher’s exact test was used to assess the potential association of clinical and CT characteristics with BHD. All analyses were conducted using SPSS version 18.0 software (IBM Corp., Armonk, NY, USA). *P*-values less than 0.05 were considered to be statistically significant.

**Table 1:** Demographic and clinical features.

	BHD (n = 6)	Non-BHD (n = 11)	*P*—value
Age (years, mean ± SE)	56.2 ± 6.8	61.4 ± 3.8	0.483
Sex (male:female)	3:3	6:5	> 0.999
Smoking history (never-smoker:smoker)	5:1	4:7	0.600
FVC (%)	94.7 ± 5.7	95.1 ± 7.8	0.967
FEV_1_ (%)	101.3 ± 7.4	91.3 ± 11.4	0.467
FVE_1_/FVC (%)	76.1 ± 3.2	72.6 ± 6.9	0.433
Proteinuria	1/5 (20.0%)	1/9 (11.1%)	> 0.999
Hematuria	1/5 (20.0%)	3/9 (33.3%)	> 0.999
Spontaneous pneumothorax	3/6 (50.0%)	1/11 (9.1%)	0.099
Family history of pneumothorax	2/6 (33.3%)	1/11 (9.1%)	0.515
Typical skin lesions*	0/4 (0.0%)	0/11 (0.0%)	N/A
Typical renal lesions^†^	0/4 (0.0%)	0/9 (0.0%)	N/A

*fibrofolliculomas or trichodiscomas

^†^renal cancer with early onset (< 50 years), or multifocal or bilateral location, or with mixed chromophobe and oncocytic histology.

Abbreviations: WBC, white blood cell; BUN, blood urea nitrogen; AST, aspartate transaminase; ALT, alanine transaminase; FVC, forced vital capacity; FEV_1_, forced expiratory volume for 1 second; BHD, Birt-Hogg-Dube syndrome; SE, standard error of mean; NA, not available

**Table 2:** Characteristics on chest computed tomography scans.

	BHD (n = 6)	Non-BHD (n = 11)	*P*-value
Number of cysts			0.728
< 20	0 (0.0%	2 (18.2%)	
20–40	0 (0.0%)	1 (9.1%)	
41–100	4 (66.7%)	7 (63.6%)	
> 100	2 (33.3%)	1 (9.1%)	
Dominant location			0.549
Central dominant	1 (16.7%)	0 (0.0%)	
Evenly distributed	2 (33.3%)	5 (45.5%)	
Peripheral dominant	3 (50.0%)	6 (54.5%)	
Size of cysts (mm)			
Median value of minimum size (IQR)	3.7 (2.1)	4.0 (1.20)	0.537
Median value of maximum size (IQR)	39.4 (11.4)	15.8 (7.8)	0.001*
CPA involvement			0.515
No	4 (66.7%)	10 (90.9%)	
Yes	2 (33.3%)	1 (9.1%)	
Multiseptated cyst			0.110
No	4 (66.7%)	11 (100.0%)	
Yes	2 (33.3%)	0 (0.0%)	
Variable morphology			0.002*
No	0 (0.0%)	9 (81.8%)	
Yes	6 (100.0%)	2 (18.2%)	

*P < 0.05, Mann-Whitney U test and Fisher’s exact test.

Abbreviations: BHD, Birt-Hogg-Dube syndrome; IQR, interquartile range; CPA, costophrenic angle.

## Results

### Demographic and clinical features

Six patients (0.06%) were diagnosed to have BHD in the basis of the *FLCN* gene study. We compared the demographic and clinical characteristics between patients in the BHD group and the non-BHD group. Age, sex, and smoking history were not significantly different between the two groups. All basic laboratory findings, routine urine analysis, and pulmonary function tests were similar between the two groups. Spontaneous pneumothorax tended to be more frequent in the BHD group (50.0%) than in the non-BHD group (9.1%), but the difference was not statistically significant (*P* = 0.099). The prevalence of family history of pneumothorax was similar between the two groups. Similarly, the prevalence of renal lesions and skin lesions was not significantly different between the groups. None of the patients in the study had a family history of typical renal or skin lesions (data not shown).

### Characteristics of chest CT scan

**Fig 2:**
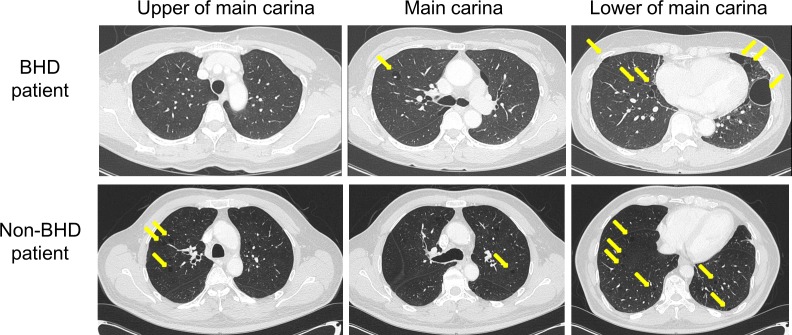
Chest CT image findings of pulmonary cysts *CT*, computed tomography; *BHD*, Birt–Hogg–Dube syndrome.

Pulmonary cysts observed on chest CT in patients with and without BHD showed markedly different characteristics (Fig 2). First, the maximum size of the cysts in the BHD group (median 39.4 mm; interquartile range [IQR] 11.4 mm) was significantly larger than that in the non-BHD group (median 15.8 mm; IQR 7.8 mm; *P* = 0.001). Second, all the patients with BHD had cysts with variable morphology whereas only 18.2% of the patients without BHD had cysts with variable morphology (*P* = 0.002). Some patients (33.3%) in the BHD group had multiseptated cysts but no cysts of this type were found in the non-BHD group (*P* = 0.110). However, there was no significant difference in the number of cysts, the dominant location of these cysts, and CPA involvement between the two groups (Table 2).

### Clinical characteristics in BHD group

Typical skin and renal lesions were not observed in our patients. However, some atypical skin and renal lesions were found as described below. The only skin lesion identified in the 4 patients who underwent a skin examination by a dermatologist was a white plaque on the nose in one patient. This lesion was identified on biopsy to be a fibroma. Of the 4 patients with renal imaging available, 2 had renal lesions, one had multiple renal cysts, and one had clear cell-type renal cell carcinoma. Only the patient with renal cell carcinoma had proteinuria and hematuria. Three patients (50.0%) had a history of pneumothorax. All patients had normal lung function tests (Table 3).

**Table 3:** Clinical and *FLCN* gene characteristics in BHD group.

No	Age	Sex	Skin lesion		Renal lesion		Renal function impairment	Lung function impairment	Pneumothorax history		FLCN gene mutation
			Typical	Atypical	Typical	Atypical			Rt	Lt	
1	43	M	None	None	None	None	None	None	0	1	c.1215C>G
2	57	F	None	Yes*	N/A	N/A	None	None	2	0	c.1285dupC
3	60	M	None	None	None	None	None	None	0	0	c.1285dupC
4	70	F	None	None	None	**Yes**^**†**^	None	None	1	0	c.1557delT
5	31	F	N/A	N/A	N/A	N/A	None	None	0	0	c.1557delT
6	76	M	N/A	N/A	None	**Yes**^**‡**^	None^§^	None	0	0	c.1285delC

*Fibroma on nose

^†^multiple renal cysts

^‡^renal cell carcinoma (clear cell type)

^§^proteinuria and hematuria.

Abbreviations: BHD: Birt-Hogg-Dube syndrome; FLCN, folliculin; N/A, not available.

### FLCN gene characteristics in BHD group

In the *FLCN* mutation study, 2 patients were found to harbor c.1285 dupC, while another 2 patients carried c.1557delT; of the remaining 2 patients, one carried c.1215C>G and the other c.1285delC (Table 3).

### Clinical and genetic features of Korean patients with

We analyzed a total of 12 Korean patients with BHD, including subjects detected in the present study (Patient No. 1–6) and those previously diagnosed with BHD in old literatures (Patient No. 7–12). The male to female ratio was 5:7, and the mean age was 50.5 ± 15.5 (31–76) years. Nine patients (75.0%) had a history of pneumothorax. None of the 9 Korean patients with BHD who underwent renal imaging had typical renal lesions (0.0%), although atypical lesions (renal cyst or clear cell renal cancer) was present in 3 patients. Two (20.0%) of the 10 patients who underwent skin examination had typical skin lesions. Atypical lesions (fibromas or papules with lymphocyte infiltration) were present in 3 patients. Among the total study population of 12 patients, nine (75.0%) had experienced pneumothorax. Patient 3 had adenocarcinoma of the lung, and underwent lung biopsy for cancerous lesions and benign cystic lesions. However, we did not find significant lung pathology related to BHD (Table 3).

The most frequently observed *FLCN* mutations were c.1285dupC (33.3%). Of the 4 patients with c.1285dupC, 2 presented typical skin lesions (50.0%). The next most frequently observed mutation was c.1557delT (25.0%). Patients 11 and 12 were related by blood to patient 10; hence, they shared the same *FLCN* mutation, c.882_884delTTC. In addition, c.1215C>G and c.1285delC were observed in each one patient. There was no significant relationship between genotype and phenotypic characteristics (Table 4).

**Table 4:** Clinical and genetic features in Korean patients with BHD.

No	Age	Sex	Typical skin lesion		Typical renal lesion		Lung lesion		FLCN gene mutation
			Typical	Atypical	Typical	Atypical	History of pneumothorax	Others	
1	43	M	None	None	None	None	Yes		c.1215C>G
2	57	F	None	**Yes†**	N/A	N/A	Yes		c.1285dupC
3	60	M	None	None	None	None	None	**Lung cancer**	c.1285dupC
4	70	F	None	None	None	**Yes§**	Yes		c.1557delT
5	31	F	N/A	N/A	N/A	N/A	None		c.1557delT
6	76	M	N/A	N/A	None	**Yes∥**	None		c.1285delC
7	31	F	None	**Yes‡**	None	None	Yes		c.1557delT
8	43	M	**Fibrofolliculoma, trichodiscoma**	None	N/A	N/A	Yes		c.1285dupC
9	54	M	**Fibrofolliculoma**	None	None	**Yes**^**¶**^	Yes		c.1285dupC
10*	40	F	None	**Yes**^**†**^	None	None	Yes		c.882_884delTTC
11*		F	None	None	None	None	Yes		c.882_884delTTC
12*		F	None	None	None	None	Yes		c.882_884delTTC

*Individuals related to each other by blood

^†^fibroma

^‡^papules with lymphocyte infiltration

^§^multiple renal cysts

^∥^renal cell carcinoma (clear cell type)

^¶^Single renal cyst.

Abbreviations: BHD, Birt-Hogg-Dube syndrome; FLCN, folliculin; N/A, not available

## Discussion

In this first prospective cohort study, we carefully reviewed the chest CT scans obtained from 10,833 patients over a period of 1 year and diagnosed 6 patients (0.06%) with BHD. Given the rarity of BHD, no previous study has attempted to detect the syndrome prospectively. Therefore, the incidence and prevalence of BHD have not yet been reported. We could not determine the prevalence and incidence of BHD in this study because at least 2 years are needed to define incidence, the research was conducted at a single institution, and the subjects were not representative of the general population. However, we can assume that many BHD patients have been underdiagnosed. The present study indicates that BHD can be detected if the cysts are actively sought during a careful review of chest CT scans.

Pneumothorax is one of main manifestations of BHD. Therefore, many studies have found BHD in patients previously diagnosed as having spontaneous pneumothorax. BHD is considered to be the cause in 5–10% of spontaneous pneumothorax cases [16,19]. However, the present study included patients with BHD who did not have such a history (50%). Careful review of the chest CT scans of patients with multiple pulmonary cysts, even those “without a pneumothorax history,” might be helpful in identifying patients with BHD and their family members. Therefore, radiologists should carefully review multiple cysts, even in patients without pneumothorax, to differentiate BHD.

In Korea, the first BHD case was reported in 2008. After the last reported case in 2012, there have been no reports on BHD since 4 years. Moreover, no Korean reviews of BHD have been published. This study found typical skin lesions in 20.0% of Korean cases. Typical skin lesions are present in approximately 90% of patients with BHD in the USA and Europe, but less frequently in Asia [8,20–23]. For example, in Taiwan, Yang et al reported that no patients with BHD presented with typical skin lesions [9]. Our study is consistent with this Taiwan study, showing a low prevalence of skin lesions (20.0%). We did not identify renal lesions in any of our Korean cases. In the USA and Europe, typical renal lesions are present in 15–35% of patients with BHD, but are less frequently encountered in Asia. Ding et al. found no renal lesions, including renal cysts [7]. Our findings are consistent with those of that Asian study. Thus, we can assume that typical skin and renal lesions are less common in Asian patients with BHD. In the USA and Europe, spontaneous pneumothorax has been reported to occur in 24–41% of cases [11,24], but has been reported in 68% of cases in China [7]. Our study also showed high prevalence of spontaneous pneumothorax (75%), consistent with the Asian study. However, we should consider the critical point that the detection rate of skin and renal lesions depends on the design of the study. For example, review articles that include many unrelated subjects detected incidentally may find a high prevalence of skin and renal lesions [20], whereas family studies including many related subjects detected by family history might show a relatively low prevalence of these lesions [7]. The prevalence of pneumothorax also depends on the design of the study. The low prevalence of skin and renal lesions and high prevalence of pneumothorax in the present study might reflect the fact that we included patients with pulmonary cysts prospectively. Further, the subjects in our study were relatively young, whereas many studies have reported that renal lesions typically manifest in older people [1]. This age-dependent nature of renal lesions may affect the low prevalence of renal lesions in our study. Importantly, the number of patients with BHD in our study is too small to reach firm conclusions regarding racial differences. Further studies comparing the prevalence of skin and renal lesions and pneumothorax according to country are needed.

Our study contained patients with BHD and atypical renal lesions. The presence of multiple renal cysts (22.2%) in Korean patients with BHD cannot be considered to be a manifestation of BHD, because the incidence of simple renal cysts in the general population is 20–30% [25]. No typical renal lesions were found in this study, consistent with a previous Chinese study [11].

Pulmonary function tests were within normal ranges in all our subjects, which is consistent with previous reports [11,26,27]. Although our patients with BHD had multiple cysts, these did not affect pulmonary function.

Our study compared the results of chest CT scans between a BHD group and a non-BHD group. Large-sized cysts and variable morphology were characteristic CT findings in the patients with BHD, which again is consistent with previous reports [18,27,28]. CPA involvement was found in 33.3% of our patients with BHD. Tobinoet al. also reported that cysts in patients with BHD were accompanied by CPA involvement in 40.5% of their patients [18]. In our study, the cysts were predominantly of the multiseptated type in the BHD group (33.3%) whereas Agarwal et al. reported that 78% of their patients with BHD had cysts with a multiseptal appearance [28]. A larger follow-up study is needed to confirm if these additional characteristics of cysts in the BHD group are significant when compared with those in the non-BHD group.

Typical renal cancer was not detected in any of our patients but lung cancer was detected in one patient (patient 3). We performed biopsy of the lung cysts in patient 3, but could not identify any pathologic findings suggesting BHD. Moreover, we could not determine if the lung cancer is associated with BHD. Further genetic analysis of this lung specimen would be useful to determine whether these lung lesions are associated with BHD. BHD should be detected early because of its malignancy potential and hereditary nature. Because the principal role of folliculin is tumor suppression, BHD is associated with cancer, including renal cell cancer and perhaps even lung cancer [29]. Further, BHD is an inherited autosomal dominant disease, so patients with BHD have a 50% chance of producing a child with the syndrome. When BHD is diagnosed, patients and their family members should be followed up regularly with a view to detecting cancer. Therefore, we recommended that the 6 newly diagnosed BHD patients should encourage their family members to attend the hospital for check-ups. This prospective study thus alerted these patients and their families to the need for BHD and cancer screening.

This study has some limitations. First, information on skin and renal lesions was not available in some patients because of their refusal to undergo further tests. Second, we did not identify a new diagnostic marker for BHD, so further studies are needed to differentiate BHD from other cystic lung diseases. Third, we need to perform a follow-up study with detailed genetic analysis in a non-BHD group to define if other genetic mutations could be related to BHD syndrome and if there are other cyst-related genetic diseases. Finally, the extent to which selection bias may have affected our results is unknown, given that all the patients were from only one center and most underwent chest CT because they had pulmonary symptoms. Therefore, the subjects in this study do not represent the general population, and the detection rate of BHD might have been overestimated.

## Conclusions

This prospective cohort study diagnosed 6 patients with BHD in the course of one year at one institution, indicating that BHD can be detected during careful review of chest CT scans.
